# Current Challenges to the Control of Echinococcosis in China

**Published:** 2018

**Authors:** Yixia CHEN, Juntao DING

**Affiliations:** 1. College of Life Science and Engineering, Northwest University for Nationalities, Lanzhou, 730030, China; 2. College of Life Science and Technology, Xinjiang University, Urumqi, P.R. China

## Dear Editor-in-Chief

Echinococcosis is a parasitic disease mainly classified into alveolar echinococcosis (AE) and cystic echinococcosis (CE) caused by *Echinococcus multilocularis* and *E. granulosus*, respectively. China is listed as one of countries with the high prevalence of echinococcosis (1), and it is epidemic in 350 counties, some of which have the co-prevalence of both AE and CE. After more than a decade, Chinese government has made a great progress in combating echinococcosis, but it is becoming clear that there is still a long way from the eradication.

In China, there are a number of risk factors associated with the transmission of two parasites, including demographics, lifestyle, and hygiene and land use practices (1). Considering these factors, we recommend the following suggestions as supplementary measures to the existing Anti-echinococcosis Action Plan (2010 - 2020).

1) Advocating local slaughter of livestock for meat production. With the improvement of road systems, animal trade activities between villages/communities in endemic areas and the outside are increasingly frequent. Additionally, weak inspection and quarantine accelerate the dissemination of echinococcosis. These factors contribute, at least to some extent, to the elevated seropositive rate of 6 - 12 yr old children in southern Xinjiang (2). Livestock for meat production are required to be slaughtered locally, and transportation of carcasses but not alive livestock is advocated. These livestock trade activities must be fitted into National Retrospective Food Safety System.

2) Construction of at least one fixed slaughterhouse in each individual villages/communities in highly prevalent areas. In China, dog-involving synanthropic cycles are primary transmission patterns and source of infection for both human CE and AE (3). At present, slaughterhouses are still unavailable in most remote villages/communities and local residents often practise home-slaughter and feed owned and stray dogs with offal, which may largely result in current high infectious rates in livestock and dogs (4). Due to low income, local residents are not willing to send their livestock to a slaughterhouse. Therefore, free slaughtering and paying for the infected liver and lungs that are collected for late destroy are also recommended.

3) Enhancement of disease surveillance and control in towns/cities. In the past, nomadic or seminomadic pastoralism was a major form of agricultural activities in endemic areas, and herdsmen were the population at the highest risk of infection (1, 3). But the situation of agricultural activities is now changing. In Tibet, Qinghai, Xinjiang and Gansu, more and more herdsmen have recently constructed their own fixed habitations with the help of the government and engage in other agricultural production activities. Moreover, Chinese government has launched National New Urbanization Plan (2014 - 2020) in 2014. It is greatly speeding up the migration of those who live in endemic areas to nearby or even distant towns/cities. Furthermore, the quantity of dogs has swiftly increased in recent years in China.

These factors may explain, to some degree, that the morbility among herdsmen, teachers and civil servants in Xinjiang is not significantly different now (5). Therefore, it is necessary to raise public awareness via multi-media nationwide, especially in towns/cities near endemic areas.
